# T Follicular Helper-Like Cells Are Involved in the Pathogenesis of Experimental Autoimmune Encephalomyelitis

**DOI:** 10.3389/fimmu.2018.00944

**Published:** 2018-05-07

**Authors:** Jun Guo, Cong Zhao, Fang Wu, Liang Tao, Chunmei Zhang, Daidi Zhao, Shuya Yang, Dongbo Jiang, Jing Wang, Yuanjie Sun, Zhuyi Li, Hongzeng Li, Kun Yang

**Affiliations:** ^1^Department of Neurology, Tangdu Hospital, Fourth Military Medical University, Xi’an, China; ^2^Department of Immunology, Fourth Military Medical University, Xi’an, China; ^3^Department of Neurology, Air Force General Hospital PLA, Beijing, China; ^4^Department of Neurology, Xi’an Children’s Hospital, Xi’an, China

**Keywords:** experimental autoimmune encephalomyelitis, multiple sclerosis, T follicular helper cells, interlukin-21, CD40 ligand

## Abstract

Multiple sclerosis (MS) and experimental autoimmune encephalomyelitis (EAE) have been proved to be T cell-mediated autoimmune diseases. Recent researches indicate that humoral immunity is also involved in the pathogenesis of these disorders. T follicular helper (Tfh) cells are critical for B cell differentiation and antibody production. However, the role of Tfh cells in MS and EAE remains unclear. Here, we found elevated frequencies of CD4^+^CXCR5^+^PD-1^+^ Tfh-like cells in both MS patients and EAE. In EAE mice, Tfh-like cells, together with B cells, were found in the ectopic lymphoid structures in spinal cords. Moreover, Tfh-like cells promoted the antibody production *via* IL-21/IL-21R and CD40 ligand/CD40 interaction and the synergy effect of STAT3 and non-canonical NF-κB signaling pathway inside B cells. Moreover, adoptive transfer of Tfh-like cells could increase the severity and delay the remission of EAE. In conclusion, our data indicate that Tfh-like cells contribute to the pathogenesis of EAE.

## Introduction

Multiple sclerosis (MS) is a chronic autoimmune disease targeting the central nervous system (CNS). Cellular immune responses are involved in the pathogenesis of MS. Specifically, myelin-specific T cells enter the CNS, attack the myelin sheath, and pave the way for infiltration of other immune cells. T helper cells, especially Th1 and Th17, are the main T cell subsets implicated in this disease (1, 2). However, recent studies hypothesize that B cells and autoantibodies also play a contributory role in this disorder (3, 4). The presence of oligoclonal bands in the cerebrospinal fluid of MS patients was first reported in the 1950s as evidence of intrathecal antibody production (5, 6). Detection of oligoclonal bands is still widely adopted as a diagnostic (7) and prognostic indicator for MS (8). Identification of myelin-specific IgG and complement in MS plaques is suggestive of cytotoxicity mediated by the antibody or complement system (9). The therapeutic effect of anti-CD20 monoclonal antibody (10, 11) and the identification of ectopic lymphoid structures (ELSs) in secondary progressive MS (12, 13) also support the significance of humoral immunity in the pathogenesis of MS. Myelin oligodendrocyte glycoprotein peptide fragment 35–55 (MOG_35–55_)-induced experimental autoimmune encephalomyelitis (EAE), one of the most widely used MS animal models, has also been shown to be a T cell driven disease (14). Several studies have identified the contribution of anti-MOG antibodies in the pathogenesis of this disease model. First of all, MOG-specific antibodies could enhance the function of CNS-resident antigen-presenting cells by directly binding and concentrating antigens to those cells (15). In addition, the therapeutic benefit of B cell depletion therapy in EAE was also accompanied by significant reduction in the titer of anti-MOG_35–55_ antibodies (16, 17). Thus, understanding the process and regulatory mechanism of antibody production in MS and EAE mice is important in designing new therapeutic strategies for treating MS.

Formation of high-affinity antibodies is largely dependent on the activity of the germinal centers (GCs) within secondary lymphoid organs (SLOs) and the help of CD4^+^ T cells (18). T follicular helper (Tfh) cells, a recently identified subset of T helper cells, are critical for B cell differentiation and antibody production. These cells express high levels of CXCR5, CD40 ligand (CD40L), and IL-21, which enable them to migrate into the GC and activate B cells (19, 20). Meanwhile, Tfh cells express high levels of surface molecules like inducible T-cell costimulator (ICOS) and programmed cell death protein 1 (PD-1). Contemporary studies indicate that overabundance of Tfh cells is responsible for autoimmune disorders with high levels of autoantibodies, such as systemic lupus erythematosus (21) and rheumatoid arthritis (22). Circulating Tfh cells have been reported to expand during the relapse phase in MS patients. This finding suggests that Tfh cells may be involved in the pathogenesis of MS (23, 24). However, the specific function and mechanism of Tfh cells and the signal pathway responsible for antibody production in this autoimmune disease has not been clearly elucidated.

In the present work, an elevated frequency of circulating Tfh-like cells and B cells was identified in MS patients undergoing relapse. Using MOG_35–55_ peptide-induced EAE as the animal model, Tfh-like cells were found to be upregulated during the course of EAE progression. The Tfh-like cells teamed up with B cells to form ELSs in the CNS. Moreover, Tfh-like cells potently boosted antibody production by B cells in an IL-21 and CD40L-dependent manner, which was attributed to the synergistic effect between the JAK/STAT3 and the non-canonical NF-κB signaling pathways. Autoantibody activates complement system and results in demyelination. Adoptive cell transfer experiment showed that MOG_35–55_-reactive Tfh-like cells increased the severity and delayed the remission of EAE *in vivo*. Taken together, these results are indicative of the involvement of Tfh-like cells in the pathogenesis of EAE.

## Materials and Methods

### Study Population

We enrolled patients with relapsing-remitting MS (RR-MS) from 2013 to 2015 who fulfilled the 2010 McDonald’s diagnostic criteria (25) in our department. A clinical relapse was defined as appearance of new neurologic symptoms and signs or deterioration of residual disability lasting for at least 24 h with an increase of EDSS over 1.0 and new lesions on MRI scanning. Remission was considered when a stable clinical status lasted for at least 30 days since the last relapse. Patients who were not receiving disease-modifying drugs during remission were enrolled. Gender- and age-matched healthy volunteers were also included as healthy controls (HCs). The study was approved by Tangdu Hospital Ethical Review Board of Fourth Military Medical University and written informed consent was obtained from all the subjects.

### Mice and EAE Induction

Six- to eight-week-old female C57BL/6J mice were purchased from the Experimental Animal Center of the Fourth Military Medical University and bred in specific pathogen-free condition. Induction of EAE was performed as previously described (26). Briefly, each mouse was anesthetized by diethyl ether and immunized with 200 µg MOG_35–55_ peptide (synthesized by Truepeptide, Shanghai). The peptide was emulsified in Complete Freund’s adjuvant (Sigma-Aldrich, St. Louis, MO, USA) containing 4 mg/mL heat-killed *Mycobacterium tuberculosis H37RA* (Difco, Detroit, MI, USA). The emulsion was injected subcutaneously into four sites at the back of each mouse. To enhance immune reaction, each mouse was given intraperitoneal injection of 200 ng pertussis toxin (Sigma-Aldrich) at day 0 and day 2 post-immunization (p.i.). Clinical symptoms were observed daily and scored as 0, no disease; 1, paralysis of the tail; 2, impaired gait or weakness of hind limb; 3, partial hind limb paralysis; 4, hind limb paralysis; 5, hind limb and partial forelimb paralysis; and 6, moribund.

### Antibodies

For mouse cell phenotype analysis, anti CD3-percp/cy5.5, anti CD4-FITC, anti CXCR5-allophycocyanin, anti ICOS-PE, and anti PD-1-PE were purchased from BioLegend (San Diego, CA, USA). Anti CD19-FITC, anti CD138-PE, anti IgD-allophycocyanin, anti CD27-percp/cy5.5, and relevant IgG isotypes were purchased from eBioscience (San Diego, CA, USA).

For human cell analysis, anti CD3-FITC, anti CD4-percp/cy5.5, anti CXCR5-allophycocyanin, anti PD-1-PE, and anti CD19-PE were purchased from BioLegend. Anti-IL-21 neutralizing antibody (eBioscience) and anti-CD40 (BioLegend) were used for functional analysis *in vitro*.

### Tissue Sampling and Immunostaining

The mice were deeply anesthetized and perfused transcardially with cold phosphate-buffered saline (PBS) followed by perfusion with 4% paraformaldehyde. The spinal cords and spleens were dissected carefully and cryo-protected in 30% sucrose. After embedded in optimal cutting temperature compound, the specimens were sectioned into 10-μm-thick sections using a cryostat microtome. For Tfh-like cells staining, sections were incubated in the dark with anti CD4-FITC (1:100; eBioscience), rabbit anti-mouse CXCR5 (1:500; Merck Millipore, Darmstadt, Germany), anti ICOS-PE (1:100; eBioscience), or anti PD-1-PE (1:100; BD Biosciences, San Jose, CA, USA) at 4°C overnight, followed by incubation with donkey anti-rabbit IgG-Cy5 (1:1000; Jackson ImmunoResearch Laboratories, West Grove, PA, USA) for 2 h at room temperature (RT). For GC and ELSs staining, sections were first incubated with rat anti-mouse CD35 (1:100; BD Biosciences) at 4°C overnight, followed by incubation in the dark with donkey anti-rat IgG-Cy5 (1:1000; Jackson ImmunoResearch Laboratories) for 2 h at RT. After CD35 staining, sections were blocked with 10% rats serum for 1 h at RT, and then incubated in the dark with anti CD4-FITC (1:100) and anti B220-PE (1:100; both from eBioscience) at 4°C overnight. Immuno-stained sections were visualized using confocal laser scanning microscopy (BX51, Olympus Corp., Tokyo, Japan).

### Isolation of Mononuclear Cells

The dissected spleens were cut into pieces and mechanically dissociated. Cell suspension was passed through a 70-µm nylon mesh. Mononuclear cells were harvested using a mouse lymphocyte separation medium (Dakewe, Beijing, China) according to the manufacturer’s instructions. For isolating CNS mononuclear cells, the dissected brains and spinal cords were digested with collagenase II (Sigma-Aldrich). Cell pellets were passed through the nylon mesh, suspended with 30% Percoll, and loaded onto 70% Percoll. Cells were centrifuged at 2,600 rpm for 20 min without acceleration and braking. Mononuclear cells were retrieved from the 30/70 interface and washed twice with PBS.

Human peripheral blood mononuclear cells were isolated *via* gradient-density centrifugation using Ficoll-Paque medium (Dakewe, Beijing, China) according to the manufacturer’s instructions. For the relapsing MS patients, blood samples were collected before the initiation of high-dose methylpredisolone pulse therapy.

### Cell Staining and Flow Cytometry

For cell surface staining, cell suspensions were incubated with fluorescent monoclonal antibodies and relevant isotype controls at an optimal dilutions for 30 min at 4°C. After incubation, the cells were washed twice with PBS containing 2% (V/V) fetal bovine serum. Flow cytometry was performed with a FACS Calibur flow cytometer (BD Biosciences). Data were analyzed using FlowJo 10.0 software.

### Autoantibody Detection

Serum MOG_35–55_-specific antibody was detected by enzyme-linked immunosorbent assay (ELISA). The 96-well microplates were pre-coated overnight with 10 µg/mL MOG_35–55_ peptide at 4°C and blocked with 3% bovine serum albumin in PBS containing 0.1% Tween-20 (PBST) for 1 h. The plates were subsequently incubated with 100 µL mouse serum (1/100 dilution) at 37°C for 1 h. Plates were washed three times with PBST and the appropriate horseradish peroxidase-conjugated goat anti-mouse IgG was added to detect the bound Ig for an hour at 37°C. After washing, the plates were colorized with tetramethylbenzidine and absorbance was read at 450 nm. The cutoff value was defined as the mean optical density value of control samples plus two SDs.

Chemiluminescent enzyme-linked immunosorbent assay (CLISA) was used to detect MOG_35–55_-specific antibody in cell culture supernatant because of the anticipated low titer of the antibody. This procedure was similar to conventional ELISA except for the substrate solution. After adding the Lumigen PS-atto substrate (Lumigen, Inc., Southfield, MI, USA), the chemiluminescence intensity was monitored using a luminescence reader (GENios, Tecan Group Ltd., Männedorf, Switzerland). The test for repeatability of this method was presented in Figure S4 in Supplementary Material.

### Cytokine Detection

The concentration of IL-21 in mouse and human serum was measured using ELISA kits [Raybiotech, Inc. for mouse (Norcross, GA, USA) and BioLegend for human] according to the manufacturers’ instructions.

### Cell Sorting and Culturing

CD19^+^ B cells and CD4^+^ T cells were respectively enriched using B cell isolation kits and CD4^+^ T cell isolation kits (both from MiltenyiBiotec, BergischGladbach, Germany) from mouse spleen according to manufacturer’s protocols. Purified CD4^+^ T cells were then consecutively incubated with allophycocyanin-conjugated anti-CXCR5 antibody (BioLegend) and anti-allophycocyanin microbeads (MiltenyiBiotec) to isolate CD4^+^CXCR5^+^ Tfh-like cells.

For cell culture experiment, 5 × 10^5^ splenic B cells from EAE or control mice were cultured alone, or with 5 × 10^5^ splenic Tfh-like cells derived from EAE mice or control mice in the presence of 1 µg/mL MOG_35–55_ alone or with anti-IL-21 (5 µg/mL) and/or anti-CD40 (50 µg/mL). An irrelevant peptide (1 µg/mL) was used as control to test the specificity of antibody production. The culture supernatant was collected 7 days later and the titer of anti-MOG_35–55_ antibody was quantified by CLISA.

To study the mechanism of IL-21 and CD40L in boosting antibody production, purified CD19^+^ B cells derived from EAE mice were cultured alone, or with recombinant IL-21 (20 ng/mL, PeproTech, Rocky Hill, NJ, USA) and/or soluble CD40 ligand (sCD40L) (20 ng/mL, PeproTech) or with IL-21, sCD40L plus Stattic (10 µmol/L, Selleck Chemicals, Houston, TX, USA). After culturing for an appropriate time (see details in Section “Results”), the cells and supernatants were harvested for Western blotting and antibody detection, respectively.

### Western Blotting Analysis

Western blotting was performed as previously described (27). Briefly, proteins were extracted using RIPA buffer (Gensharebio, Xi’an, China). Protein concentration was measured with a Pierce BCA Protein Assay kit (Thermo Fisher Scientific, Waltham, MA, USA). Equal quality of protein was loaded in 10% SDS-PAGE gel, transferred onto nitrocellulose membranes and probed with primary antibodies. The primary antibodies employed were Bcl-6 (BioLegend), CXCR5 (EMD Millipore, Billerica, MA, USA), IL-21 (Abcam, Cambridge, UK), STAT3, Phospho-STAT3, iKK-α, Phospho-iKKα/β, NF-κB-inducing kinase (NIK), P100/52, and B lymphocyte-induced maturation protein-1 (Blimp-1) (all from Cell Signaling Technology, Danvers, MA, USA).

### Adoptive Cell Transfer

C57BL/6J mice were immunized with MOG_35–55_ to induce EAE, as described above. The donor mice were sacrificed and splenocytes were harvested at day 18 p.i. The splenocytes were cultured for 3 days in the presence of 10 µg/mL MOG_35–55_. CD19^+^ B cells and CD4^+^CXCR5^+^ Tfh-like cells were then magnetically isolated. B cells (2 × 10^6^) and/or Tfh-like cells (2 × 10^6^) were injected into pre-immunized recipient mice through the angular vein under microscope at day 2 p.i. The disease score was measured daily in a double blind manner.

### Data Analysis

All statistical analyses were performed with the SPSS19.0 software (SPSS Inc., USA). For demographic and clinical features of subjects enrolled in this study, data are presented as number with percentages or median with range. Comparisons among relapsing MS, remitting MS patients, and HCs were analyzed by Kruskal–Wallis *H* test (age), Mann–Whitney *U* test (onset age, disease duration, and EDSS) and Fisher’s exact test (percentage of female). For other statistical analysis, one-way-ANOVA was used to calculate the significance level among multiple groups. Spearman rank analysis was used to assess the correlation between two variables. A *P* value <0.05 was considered to be statistically significant. GraphPad Prism 5 (GraphPad Software, Inc., USA) was used to draw the figures.

## Results

### Demographic and Clinical Characteristics of Patients With RR-MS and HCs

As shown in Table 1, we enrolled 13 relapsing MS patients, 15 remitting MS patients, and 20 HCs. There were no difference in the percentage of female and age at sampling time point among the relapsing MS, remitting MS patients, and HCs. No statistical differences were also observed in the onset age and disease duration between the relapsing MS and remitting MS patients. In parallel to disease activity, the EDSS score was significantly higher in the relapsing MS patients than remitting MS patients (*P* < 0.001).

**Table 1:** Demographic and clinical characteristics of relapsing-remitting MS patients and HCs.

	Relapsing MS (*n* = 13)	Remitting MS (*n* = 15)	HCs (*n* = 20)	*P*value
Female, no. (%)	10 (76.9)	11 (73.3)	14 (70.0)	1.000
Age (years)	32 [21–59]	35 [21–57]	32 [20–57]	0.634
Onset age (years)	31 [19–57]	31 [20–53]	NA	0.595
Disease duration (years)	2.2 [1–7]	3 [1–8]	NA	0.658
EDSS	3.5 [1.5–5]	1.5 [0–3.5]	NA	<0.001

*MS, multiple sclerosis; HCs, healthy controls; EDSS, Kurtzke’s Expanded Disability Status Scale; NA, not applicable*.

*Data are presented as number (percentages) or median [range]. Age (years) refers to age at sampling time point. Disease duration (years) refers to interval from disease onset to sampling. Comparisons among relapsing MS, remitting MS patients, and HCs were analyzed by Kruskal–Wallis *H* test (age), Mann–Whitney *U* test (onset age, disease duration, and EDSS), and Fisher’s exact test (female ratio). A *P* value of <0.05 was considered statistically significant*.

### Upregulated Levels of Circulating Tfh-Like and B Cells in Patients With RR-MS During the Relapsing Phase

**Figure 1:**
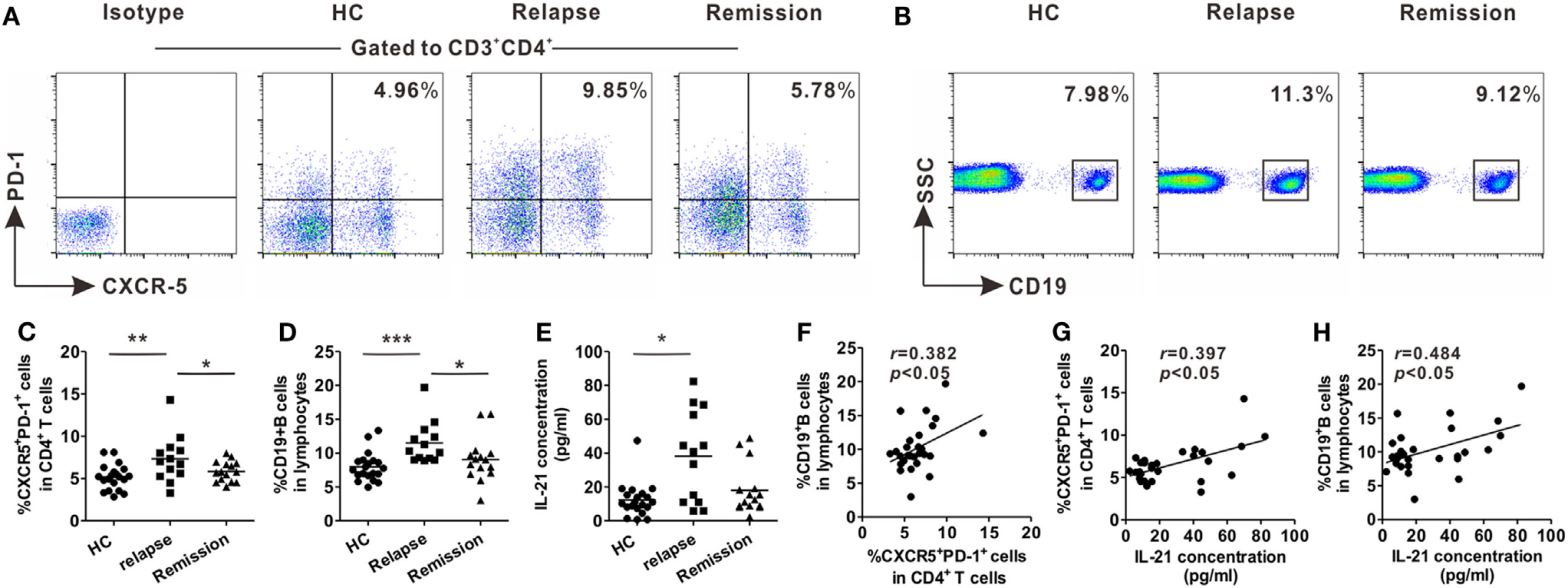
Increases in the percentages of circulating T follicular helper (Tfh)-like and B cells in relapsing-remitting MS (RR-MS) patients during the relapsing phase of the disease. Representative flow cytometry results of CD3^+^CD4^+^CXCR5^+^PD-1^+^ Tfh-like cells **(A)** and B cells **(B)** in healthy controls (HCs), relapsing and remitting patients. Numbers in the upright corner illustrated the percentage of CXCR5^+^PD-1^+^ cells in CD4^+^ T cells and the percentage of CD19^+^ B cells in lymphocytes. Comparisons of the frequencies of Tfh-like cells **(C)** and B cells **(D)** among HCs, relapsing patients and remitting patients. **(E)** Comparison of serum IL-21 level among HCs, relapsing patients, and remitting patients. **(F)** The correlation analysis between the frequency of Tfh-like cells and the frequency of B cells in patients with RR-MS. **(G)** The correlation analysis between the frequency of Tfh-like cells and serum IL-21 level in patients with RR-MS. **(H)** The correlation analysis between the frequency of B cells and serum IL-21 level in patients with RR-MS. Each data point represents an individual subject. The horizontal lines represent the mean values. HCs: *n* = 20; relapsing patients, *n* = 13; remitting patients, *n* = 15. *P < 0.05, **P < 0.01, ***P < 0.001.

Blood samples were collected from relapsing patients, remitting patients, and age- and gender-matched HCs for evaluation of the changes in frequencies of circulating Tfh-like and B cells in RR-MS, the most common form of MS. Peripheral blood mononuclear cells were isolated and stained with fluorescence-conjugated monoclonal antibodies. Circulating Tfh-like cells were defined as CD3^+^CD4^+^CXCR5^+^PD-1^+^, as previously reported (28). The percentage of circulating Tfh-like cells was higher in the relapsing patients, compared with remitting patients and HCs (Figures 1A,C). The level of B cells was also upregulated during the relapsing phase of the disease (Figures 1B,D). The level of B cells was positively correlated with the level of circulating Tfh-like cells (Figure 1F, *r* = 0.382, *P* < 0.05). The serum level of IL-21, a pivotal cytokine secreted mainly by Tfh cells, was also significantly upregulated in relapsing patients (Figure 1E). There was a positive correlation between the level of circulating Tfh-like cells and the level of IL-21 (Figure 1G, *r* = 0.397, *P* < 0.05). In addition, a significant correlation was also identified between the level of IL-21 and the level of B cells (Figure 1H, *r* = 0.484, *P* < 0.05). Our data showed Tfh-like cell, B cells, and IL-21 were altered during various phases of MS, suggesting that Tfh-like cell, B cells, and IL-21 might be involved in this chronic autoimmune disease.

### Expansion of Tfh-Like Cell in the SLOs of Mice With EAE

**Figure 2:**
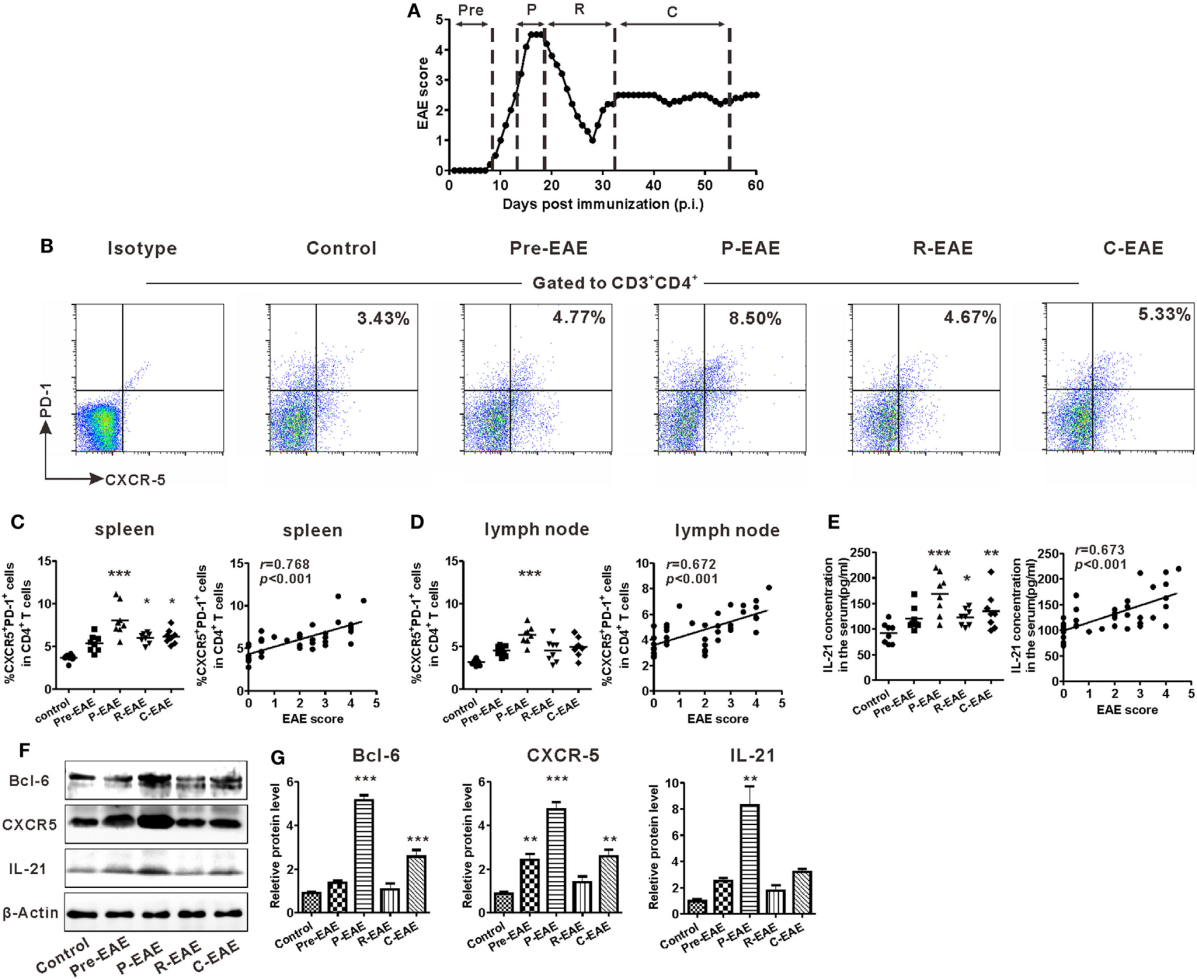
Kinetics of T follicular helper (Tfh)-like cell in the secondary lymphoid organs (SLOs) during the different phases of experimental autoimmune encephalomyelitis (EAE). **(A)** The clinical course of MOG_35–55_-induced EAE in C57BL/6J mice. Mean clinical score is shown (*n* = 8). **(B)** Representative flow cytometry results of CD3^+^CD4^+^CXCR5^+^PD-1^+^ Tfh-like cells in the spleens of EAE mice during different clinical phases. Gates were set on CD3^+^CD4^+^ cells. Numbers in the upright corner illustrated the percentage of CXCR5^+^PD-1^+^ cells in CD4^+^ T cells. Comparisons of the frequency of Tfh cells in spleens [**(C)** left] and draining lymph nodes [**(D)** left] at different phases of EAE mice (*n* = 8/time point). The correlation analysis between the EAE score and the frequency of Tfh-like cells in spleens [**(C)** right] or draining lymph nodes [**(D)** right]. **(E)** The serum level of IL-21 at different phases of EAE mice (left, *n* = 8/time point) and its correlation with EAE score (right). **(F)** Representative blots band of Bcl-6, CXCR5, and IL-21 in splenocytes of EAE mice at different phases. **(G)** Statistical data of the relative protein level of Bcl-6, CXCR5, and IL-21 in splenocytes of EAE mice at different phases. Values are mean ± SEM. For **(C–E)**, each data point represents an individual subject and the horizontal lines represent the means (*n* = 8). Results are representative of three independent experiments. Abbreviations: Pre, pre-clinical; P, peak; R, remission; C, chronic. *P < 0.05, **P < 0.01, ***P < 0.001 vs. control.

The EAE model was induced in C57BL/6J mice to study the potential role of Tfh cells in CNS inflammation. According to the disease characteristics of this model, the clinical course of EAE was divided into four phases according to the days post-immunization (dpi): pre-EAE phase (6–9 dpi, score 0–0.5), peak phase (12–20 dpi, score 4–5), remission phase (25–32 dpi, score 1–2), and chronic phase (35–60 dpi, score 1–3) (Figure 2A). The kinetics of Tfh-like cell expansion in the spleen and draining lymph nodes was examined during the different phases of EAE. The percentage of CD3^+^CD4^+^CXCR5^+^PD-1^+^ Tfh-like cells increased prior to the manifestation of clinical symptoms, reached a maximum at the peak phase, and declined during the remission phase of the disease. During the chronic phase, the percentage of Tfh-like cells increased slightly (Figures 2B–D). Similar results were achieved when CD3^+^CD4^+^CXCR5^+^ICOS^+^ was employed as the alternate marker of Tfh-like cells (Figure S1 in Supplementary Material). Changes in the serum level of IL-21 were similar to those of Tfh-like cells (Figure 2E). The protein levels of three specific molecular markers of Tfh cells, Bcl-6, CXCR5, and IL-21, also altered along with the clinical course of the disease (Figures 2F,G). Both the percentage of Tfh-like cells and the serum IL-21 level were positively correlated with the disease scores of EAE (Figures 2C–E, “*r* = 0.768, *P* < 0.001” for Tfh-like cells in spleens, “*r* = 0.672, *P* < 0.001” for Tfh-like cells in lymph nodes, “*r* = 0.673, *P* < 0.001” for IL-21). Collectively, the results indicate that Tfh-like cells as well as IL-21 varied along with the course of the disease and have a positive correlation with disease severity of EAE.

### Increased B Cells and Structure of GCs in EAE Mice

**Figure 3:**
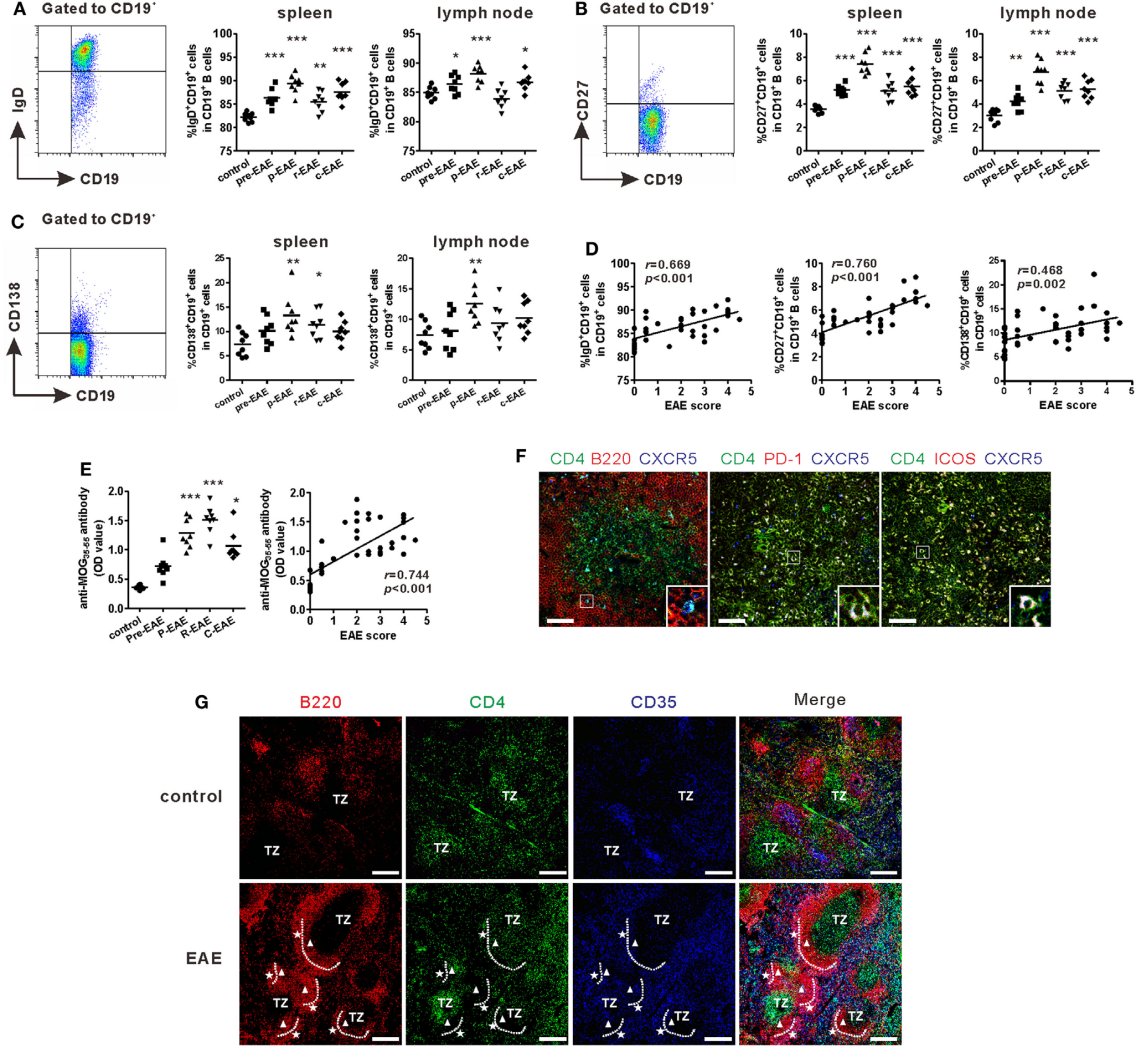
B cell profile and morphology of germinal centers (GCs) in experimental autoimmune encephalomyelitis (EAE) mice. Comparisons of the frequencies of CD19^+^IgD^+^ mature B cells **(A)**, CD19^+^CD27^+^ memory B cells **(B)**, and CD19^+^CD138^+^ plasma cells **(C)** in the spleen and draining lymph nodes at different phases of EAE mice. **(D)** The correlation analysis between each of the three subsets in the spleen mentioned above and EAE score, respectively. **(E)** The serum level of anti-MOG_35–55_ antibodies (represented as OD value) at different phases of EAE mice and its correlation with EAE score. For **(A–E)**, each data point represents an individual subject and the horizontal lines represent the means (*n* = 8/time point). Results are representative of three independent experiments. Abbreviations: Pre, pre-clinical; P, peak; R, remission; C, chronic. *P < 0.05, **P < 0.01, ***P < 0.001 vs. control. **(F)** Immunofluorescence staining of the spleen of EAE mice showed T follicular helper-like cells located at the T-B border (bar = 50 μm). **(G)** Immunofluorescence staining compared the GC structures between the spleen of control and EAE mice (TZ = T-cell zone, white triangle: dark zone, white pentacle: light zone, bar = 200 μm). Sections are representative of three mice analyzed.

Previous studies showed that Tfh cells were critical for promoting B cells proliferation, maturation, activation, and differentiation (19, 20). Accordingly, the subsets of B cells in the SLOs of EAE mice were examined. Flow cytometry results showed that the percentages of mature B cells (CD19^+^IgD^+^), memory B cells (CD19^+^CD27^+^), and plasma cells (CD19^+^CD138^+^) increased during the course of EAE and reached a maximum at the peak phase of the disease (Figures 3A–C). The percentages of all the three B cell subsets in the spleen were strongly correlated with the disease score (“*r* = 0.669, *P* < 0.001” for mature B cells, “*r* = 0.760, *P* < 0.001” for memory B cells, “*r* = 0.468, *P* = 0.002” for plasma cells) (Figure 3D). The concentration of serum anti-MOG_35–55_ increased significantly at the onset of EAE, but maximized at the remission phase; this may be partly attributed to the lag of antibody production (Figure 3E). Moreover, the titer of serum anti-MOG_35–55_ also had a positive correlation with disease score (Figure 3E, *r* = 0.744, *P* < 0.001).

Humoral immunity largely depends on the reaction within the GCs, where B cells undergo somatic hyper-mutation, class switching, and high-affinity antibody production. The morphology of GCs within the spleens of EAE and control mice was examined, respectively. Compared with control mice, the spleen of EAE mice at the peak disease phase was significantly enlarged (Figure S2A in Supplementary Material). The present results also confirmed that CD4^+^CXCR5^+^ Tfh-like cells were located at the T-B border (Figure 3F). After MOG_35–55_ challenge, CD4^+^ T cells, B220^+^ B cells, and CD35^+^ follicular dendritic cells all dramatically expanded compared with the control mice. The follicular dendritic cells in EAE mice also migrated to the periphery of the B cell zone and contributed to the formation of light zone of GCs, where B cells experienced affinity selection with the help of Tfh and follicular dendritic cells (Figure 3G; Figure S2B in Supplementary Material). By contrast, no obvious structures resembling GCs were observed in the control mice (Figure 3G; Figure S2B in Supplementary Material).

### Fate of Tfh-Like and B Cells in the CNS

**Figure 4:**
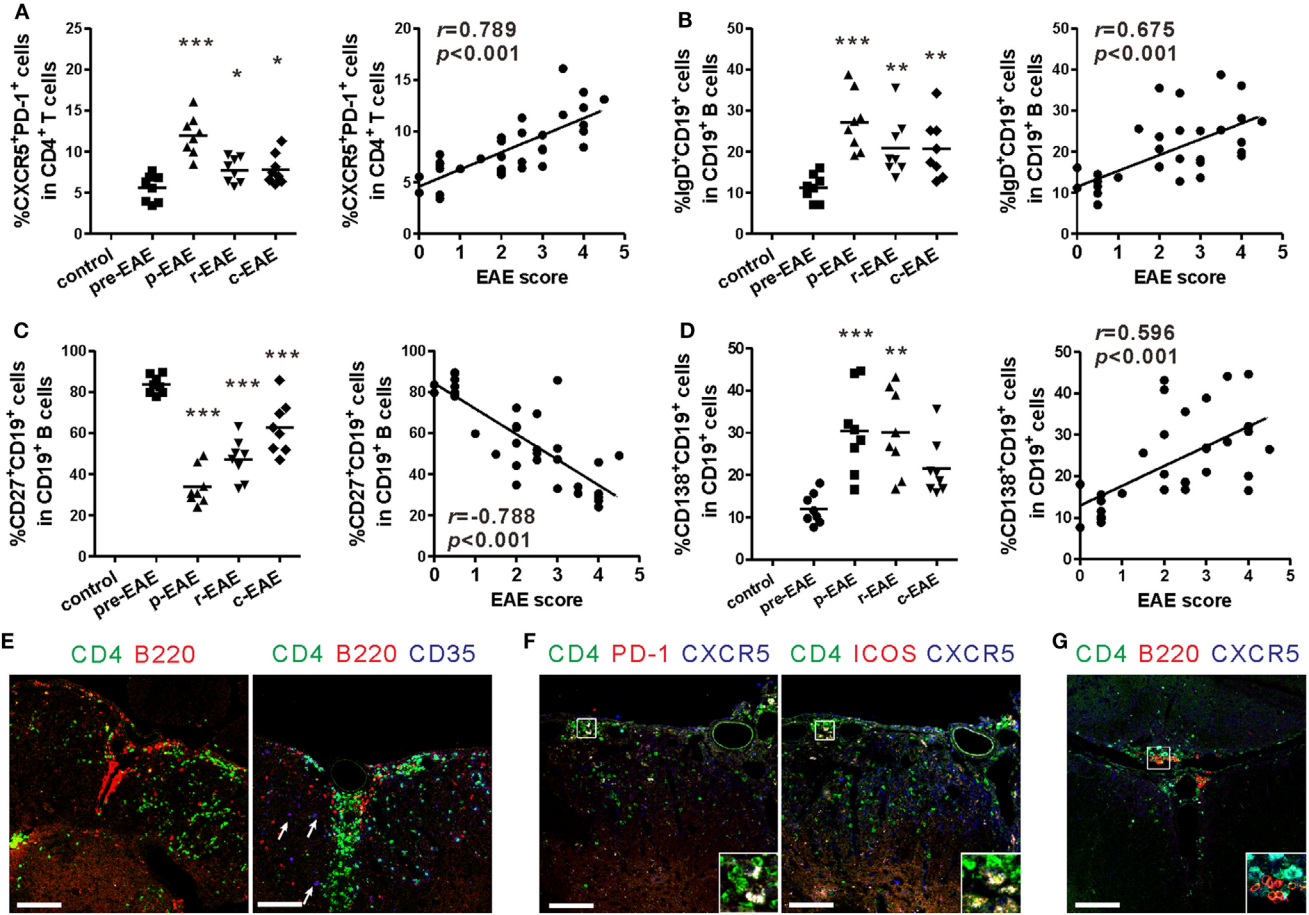
T follicular helper (Tfh)-like and B cells entering into the central nervous system (CNS). **(A)** Comparison of the frequency of Tfh-like cells in the CNS (spinal cord and brain) of experimental autoimmune encephalomyelitis (EAE) mice at different phases (left) and its correlation with EAE score (right). **(B)** Comparison of the frequency of mature B cells in the CNS (spinal cord and brain) of EAE mice at different phases (left) and its correlation with EAE score (right). **(C)** Comparison of the frequency of memory B cells in the CNS (spinal cord and brain) of EAE mice at different phases (left) and its correlation with EAE score (right). **(D)** Comparison of the frequency of plasma cells in the CNS (spinal cord and brain) of EAE mice at different phases (left) and its correlation with EAE score (right). For **(A–D)**, each data point represents an individual subject and the horizontal lines represent the means (*n* = 8/time point). Results are representative of three independent experiments. Abbreviations: Pre, pre-clinical; P, peak; R, remission; C, chronic. *P < 0.05, **P < 0.01, ***P < 0.001 vs. Pre. **(E)** ELSs and germinal center (GC)-like structures forming in the spinal cords of EAE mice at peak phase (12–20 dpi, score 4–5). Arrows show CD35^+^ follicular dendritic cells. **(F)** Tfh-like cells infiltrated into the spinal cords of EAE mice at peak phase (12–20 dpi, score 4–5). **(G)** Tfh-like cells co-localized with B cells in the spinal cords of EAE mice at peak phase (12–20 dpi, score 4–5). Sections are representative of three mice analyzed. Bar = 100 μm.

The aforementioned results indicate that the percentages of peripheral Tfh-like and B cells are strongly correlated with the severity of EAE. Because infiltration of mononuclear cells into the CNS is one of the key characteristics of the EAE model, the fate of Tfh-like and B cells in the CNS during the various stages of EAE was subsequently analyzed. The percentages of Tfh-like, mature B, and plasma cells in the CNS all reached a maximum at the peak phase of EAE, and were all positively correlated with the disease score (Figures 4A,B,D, “*r* = 0.789, *P* < 0.001” for Tfh-like cells, “*r* = 0.675, *P* < 0.001” for mature B cells, “*r* = 0.596, *P* < 0.001” for plasma cells). Surprisingly, the percentage of memory B cells within the CNS peaked at the pre-clinical phase, rapidly dropped to a minimum when the disease score was the highest, and increased again during the remission and chronic phases. These features were entirely different from those within the SLOs (Figure 4C).

In the immunofluorescent analysis of the spinal cords, CD4^+^ T cells and B cells co-localized within the inflammatory areas to form an ELS (Figure 4E, left). Infiltration of CD35^+^ follicular dendritic cells could have contributed to the formation of GC-like structures in the spinal cords of EAE mice (Figure 4E, right). Similarly, a number of CD4^+^CXCR5^+^PD-1^+^ and CD4^+^CXCR5^+^ICOS^+^ Tfh-like cells were also present within the inflammatory area of the spinal cords of EAE mice (Figure 4F). Some B cells were in intimate contact with Tfh-like cells, which could have provided activation signals to the B cells (Figure 4G). In areas where B lymphocytes aggregated, decrease in the quantity of the myelin protein MOG was observed, suggesting the aggressiveness of B cells to the myelin sheath (Figure S3A in Supplementary Material). In addition, the complement component C3b was deposited at sites where there were substantial losses in MOG protein. The C3b was surrounded extensively by CD19^+^ B cells. Based on this observation, activation of the classical complement system by antigen-antibody complexes may be involved in the inflammation of the CNS (Figure S3B in Supplementary Material).

### Tfh-Like Cells Help B Cells Produce Anti-MOG_35–55_ *via* IL-21 and CD40L

**Figure 5:**
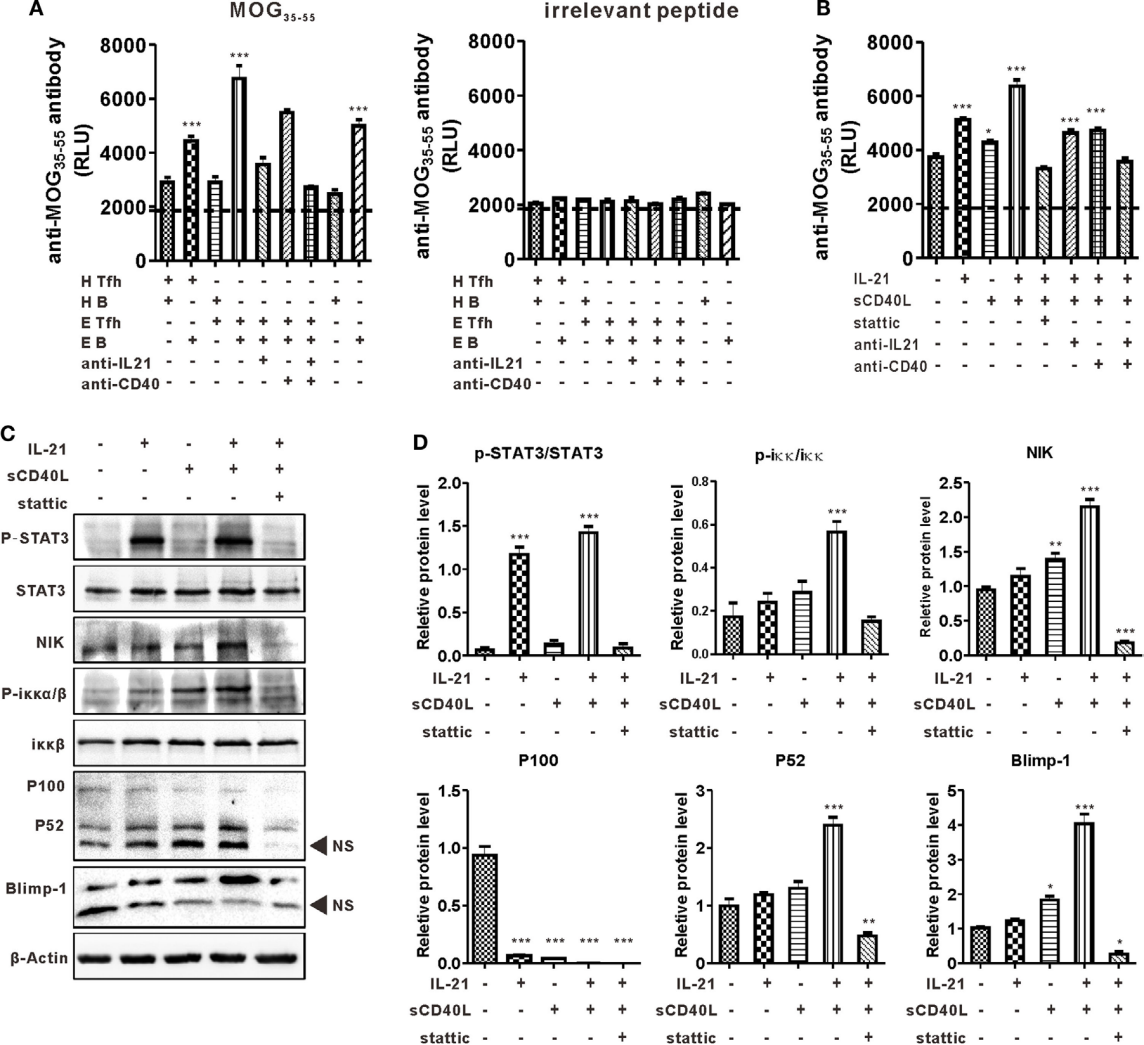
T follicular helper (Tfh)-like cells help B cells product autoantibody in an IL-21 and CD40 ligand (CD40L)-dependent manner. **(A)** Tfh-like and B cells from healthy controls (HCs) and experimental autoimmune encephalomyelitis (EAE) mice were cocultured. The level of anti-MOG_35–55_ antibodies were measured by chemiluminescent enzyme-linked immunosorbent assay (CLISA) in the supernatant of Tfh/B coculturing system. H Tfh-like and H B stand for Tfh-like from HCs and B cells from HCs, respectively. E Tfh-like and E B stand for Tfh-like from EAE mice and B cells from EAE mice, respectively. ***P < 0.001 vs. the first lane. **(B)** Sorted B cells from EAE mice were stimulated with MOG_35–55_ and relevant cytokines or inhibitors for 7 days. The levels of anti-MOG_35–55_ antibodies in the supernatant were measured by CLISA. *P < 0.05, ***P < 0.001 vs. the first lane. **(C)** Sorted B cells from EAE mice were subjected to the indicated culture conditions. Cells were stimulated for 3 h and then lysed for western blotting analysis to detect the level of the indicated protein markers except B lymphocyte-induced maturation protein-1 (Blimp-1). For Blimp-1, B cells were cultured for 48 h and then prepared for western blotting analysis. NS, nonspecific band. **(D)** Statistical data of the relative protein level. Data were shown as mean ± SEM. *P < 0.05, **P < 0.01, ***P < 0.001 vs. the first lane. Results are representative of three independent experiments.

Co-localization of Tfh-like and B cells in the CNS suggested the disease-promoting effect of these cells in the present murine MS model, so a cross coculturing system was established to further understand the interaction between these two kinds of cells (see details in Materials and Methods). The level of anti-MOG_35–55_ antibody in the culture supernatant was detected by CLISA and represented by the “relative light unit.” Results showed that B cells from EAE mice secreted a certain level of anti-MOG_35–55_ antibody, while those from control mice did not undergo efficient antibody production when cultured alone or cocultured with Tfh-like cells. The titer of anti-MOG_35–55_ was much higher when B cells, regardless from EAE or normal mice, were cocultured with EAE Tfh-like cells than that of B cells coculturing with control Tfh-like cells. This suggested that Tfh-like cells from EAE mice, compared with those from control mice, had a much greater potential in boosting the antibody production capacity of B cells (Figure 5A, left). Previous studies reported that IL-21 and CD40L were the most critical functional molecules of Tfh-like cells (20). Hence, blocking antibodies of IL-21 and CD40 were added into the culture system to further verify the function of these two molecules in the present model. The titer of anti-MOG_35–55_ antibody slightly decreased in the presence of anti-CD40 antibody, while anti-IL-21 neutralizing antibody significantly reduced the production of anti-MOG_35–55_. A highly significant reduction in the titer of anti-MOG_35–55_ was observed when anti-IL-21 and anti-CD40 were used simultaneously (Figure 5A, left). The antigen specificity in the reaction between Tfh-like and B cells was validated by using an irrelevant peptide as control (Figure 5A, right).

The mechanism of IL-21 and CD40L in promoting antibody production was investigated in detail. Purified B cells from EAE mice were cultured alone or with IL-21 and/or sCD40L. When used alone, IL-21 significantly upregulated anti-MOG_35–55_ production, while sCD40L only slightly stimulated the production of anti-MOG_35–55_. The level of anti-MOG_35–55_ was extensively increased when IL-21 and sCD40L were used in combination, suggesting the synergistic effect of these two proteins. When Stattic, a STAT3 phosphorylation inhibitor, was added to the culture system, the antibody-promoting effect of IL-21 and CD40L was almost completely inhibited and the titer of anti-MOG_35–55_ was even lower than that of the control group (Figure 5B). Theoretically, inhibiting the phosphorylation of STAT3, the main transcriptional factor downstream of IL-21 receptor, should not affect the function of CD40L. However, in the present study, Stattic downregulated the production of anti-MOG_35–55_ to a degree that was even lower than the baseline level. This observation suggests the existence of a STAT3-linked crosstalk between the JAK/STAT3 axis and the non-canonical NF-κβ pathway, the downstream signaling pathways of IL-21 receptor and CD40L. Western blotting analysis showed that IL-21 alone activated the phosphorylation of STAT3 but CD40L–CD40 ligation did not. As expected, the non-canonical NF-κB pathway was activated by sCD40L, which was represented by elevated levels of NIK, p-IKKα/β, and p52. Surprisingly, IL-21, which only activates JAK/STAT3 axis theoretically, also stimulated the non-canonical NF-κB pathway. Stimulation was more distinct when sCD40L was added simultaneously. More importantly, IL-21 and sCD40L synergistically upregulated the expression of Blimp-1, the master transcription factor that regulates plasma cell differentiation. Inhibition of the aforementioned synergistic effects by Stattic is indicative of the pivotal role of p-STAT3 in linking these two pathways (Figures 5C,D).

### Adoptive Transfer of Tfh-Like or B Cells Delayed Remission of MOG_35–55_-Induced EAE

**Figure 6:**
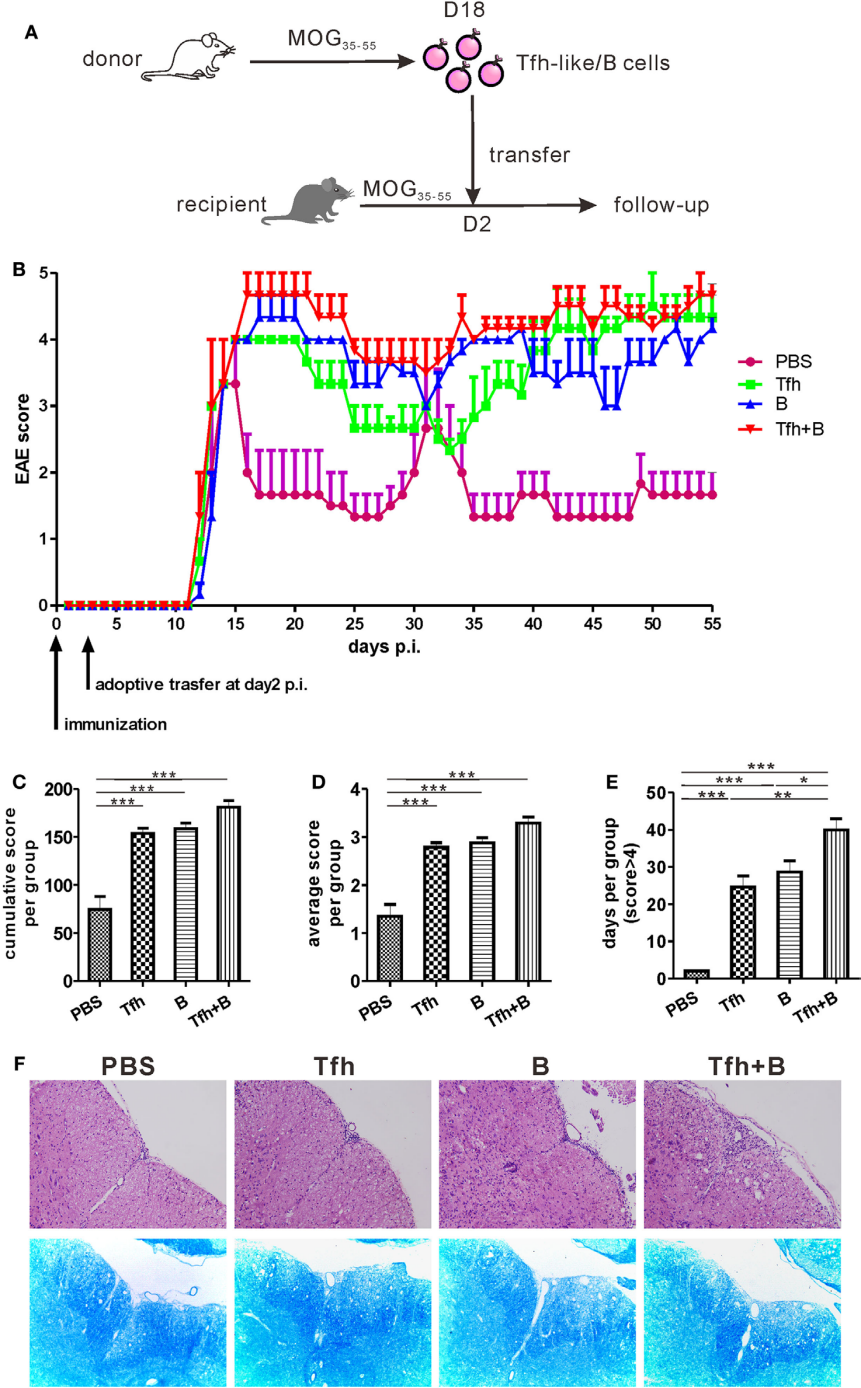
Adoptive transfer of T follicular helper (Tfh) or B cells delayed remission of MOG_35–55_-induced experimental autoimmune encephalomyelitis (EAE). **(A)** The process diagram of adoptive transfer experiments. Briefly, CD4^+^CXCR5^+^ Tfh-like cells and CD19^+^ B cells were, respectively, sorted from the spleens of MOG_35–55_-immunized mice (donor) at 18 dpi. Isolated CD4^+^CXCR5^+^ T cells and/or CD19^+^ B cells were then transferred into pre-immunized mice (recipient) at 2 dpi. **(B)** The EAE score were measured in the following 55 days after antigenic challenge (*n* = 3 mice/group). **(C)** Cumulative score of each mouse in the 55 days. **(D)** Average score of each mouse in the 55 days. **(E)** The number of days for each mouse to score more than 4. All data were shown as mean ± SEM. *P < 0.05, **P < 0.01, ***P < 0.001. **(F)** H&E and LFB staining for the spinal cords of the adoptive transferred mice.

To verify the potential pathogenic roles of Tfh-like and B cells *in vivo*, Tfh-like and/or B cells were transferred from MOG_35–55_-immunized mice into recipient mice 2 days after the recipient mice were immunized with MOG_35–55_ in complete Freund’s adjuvant (Figure 6A). The disease status of the recipient mice was investigated over the subsequent 55 days. As shown in Figure 6B, adoptive transfer of Tfh-like and/or B cells did not accelerate the onset of EAE. Compared with PBS-treated mice, mice that received adoptive transfer showed a significant delay in disease remission. Mice that were co-transferred with Tfh-like and B cells showed almost no indication of remission (Figure 6B). The three groups of mice that underwent adoptive cell transfer had an elevated cumulative (Figure 6C) and average disease score (Figure 6D) compared with the control group. But no statistical differences could be identified among the three adoptive cell transfer groups. Cotransferring with Tfh-like and B cells resulted in arresting disease progression in the most serious conditions; that is, there were more days in which the disease score was greater than 4 in this group when compared to the other three groups (Figure 6E). We also detected the titers of anti-MOG_35–55_ antibodies in the recipient mice and found that transferring Tfh-like and B cells separately or simultaneously could significantly increase the titers of anti-MOG_35–55_ antibodies (Figure S5 in Supplementary Material). Moreover, disease exacerbation in the mice that received adoptive cell transfer was accompanied by aggravated inflammatory and demyelinating lesions in the spinal cords (Figure 6F). Collectively, these results suggest that Tfh-like cells collaborated with B cells in the pathogenesis of EAE, possibly through increasing the severity of disease and delaying the remission of inflammation within the CNS.

## Discussion

Despite the fact that MS is a T cell-dominant autoimmune disorder, humoral immunity may also be involved in the pathogenesis of this disease. However, the mechanism that activates B cells remains ambiguous. Tfh cells have recently been identified as the most important activator of humoral immune response; excessive Tfh responses are believed to result in autoimmunity (21, 22). In the present study, circulating CD4^+^CXCR5^+^PD-1^+^ Tfh-like cells were identified from the peripheral blood of MS patients, the extent of which was positively correlated with circulating B cells and disease activity. These findings are consistent with previous studies (23, 24), and prompted out quest for the ultimate role of Tfh cells in this disease. In this work, the involvement of Tfh-like cells was validated in EAE, the murine model of MS; these cells enhance autoantibody production and are involved in the pathogenesis of EAE.

A strong association between aberrant Tfh response and autoimmunity has been identified in both humans and experimental animal models (29, 30). However, most of the researches to date were conducted on spontaneous disease models, which are primarily mediated by genetic mutations. The change and function of Tfh cells in antigen-challenged autoimmune disease models have not been investigated. In the present study, dynamic changes of Tfh-like and B cells in the SLOs of MOG_35–55_-induced EAE were examined. The percentage expressions of both Tfh-like and B cells in these sites were highly correlated with disease activity. Infiltration of leukocytes into the target organ is a key characteristic of autoimmune diseases. In the present work, infiltration of Tfh-like and B cells was observed in the CNS during the clinical course of EAE. Similar to the SLOs, increases in the percentages of Tfh-like cells, IgD^+^ mature B cells, and CD138^+^ plasma cells in the CNS were positively correlated with disease score. However, the number of CD27^+^ memory B cells in the CNS peaked at the pre-clinical phase and declined to a minimum during the peak phase of EAE, which was very different from what was identified in the SLOs. A probable explanation for this discrepancy is that MOG_35–55_-specific memory B cells infiltrated into the CNS prior to the onset of clinical symptoms and directly differentiated into plasma cells with the help of CNS-infiltrated Tfh-like and follicular dendritic cells. Recently, many studies focusing on regional immunity have found that the function and phenotype of immune cells in tissues, such as gut (31), liver (32), and adipose tissue (33), differ from those in lymphoid organs (34, 35). But, up to now, there is still no study comparing the difference between lymphocytes from the CNS and peripheral lymphoid organs. It may be of great significance to compare the phenotype and potency of CNS-derived Tfh/B cells and SLOs-derived Tfh/B cells. To study the characteristics of the CNS-derived Tfh and B cells may help us further understand the role of these cells in MS and EAE.

Apart from causing intense inflammation, infiltrated leukocytes often aggregate together and form organized clusters in the form of ELSs (36). Usually, T cells, B cells, dendritic cells, and local resident cells such as macrophages and stromal cells can form clusters within the inflamed tissues (36). ELSs, which was formed by infiltrated T and B cells, have been identified in the meninges of secondary progressive MS patients and EAE mice, suggesting the presence of chronic inflammation and persistent immunopathological processes within the CNS of subjects suffering from these disease entities (12, 13, 37). In the present animal disease model, co-localization of CD4^+^ T cells and B200^+^ B cells was observed in the spinal cords of EAE mice, forming ELSs. In addition, GC-like structures, which are characterized by the infiltration of CD35^+^ follicular dendritic cells, could be identified within the inflammatory lesions of the CNS. These structures may serve as rendezvous sites wherein immunologic responses such as antigen representation and antibody affinity maturation are initiated adjacent to autoantigens and target organs. Recent studies have demonstrated the importance of Tfh cells or Tfh-associated markers in the formation and maintenance of ELSs (37–39). In the present work, the existence of CD4^+^CXCR5^+^PD-1^+^ Tfh-like cells within the ELSs was also confirmed. These Tfh-like cells were in close contact with B cells, enabling the latter to receive activation signals from the Tfh-like cells. Peters and colleagues have demonstrated in a previous study that adoptive transfer of MOG-specific 2D2 Th17 cells into wild-type recipients could induce ELSs within the CNS (37). Flow cytometry data revealed that these so-called Th17 cells co-expressed markers for Th17 (CCR6) and Tfh cells (CXCR5, ICOS, Bcl-6); the results suggest that these transferred cells also acquire some features of Tfh cells in the CNS. Previous studies on T cell plasticity indicate that Th1, Th2, or Th17 cells can acquire the phenotype and function of Tfh cells during their differentiation (40–42). Given the assumption that EAE is a Th17-dominated disease, is it possible for a group of the CNS-infiltrated Th17 cells to transform into Tfh cell and form ELSs? It is unclear at this stage whether there is a specific driving force that accounts for this transformation within the CNS. Further studies are required to clarify the interactions among local resident cells, cytokines, chemokines, and the infiltrated T cells.

**Figure 7:**
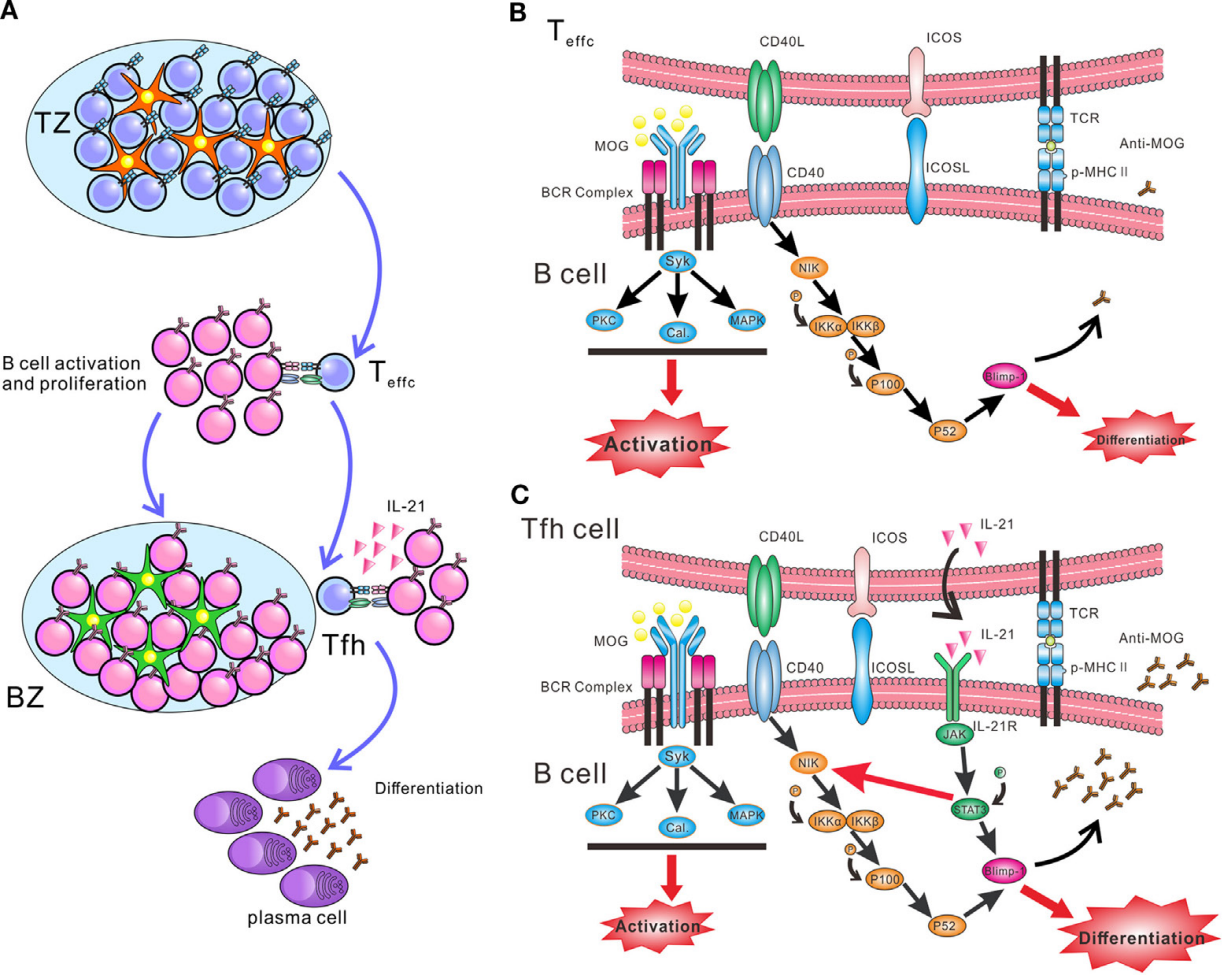
Schematic diagram of interaction between T follicular helper (Tfh) and B cells initiating synergetic effect of IL-21/IL-21R and CD40 ligand (CD40L)/CD40 to promote plasma cell differentiation and antibody production in EAE. **(A)** The process of antigen-activated T cells and B cells migrating to the B cell follicles, where enables B cells receive helper signals from Tfh cells. Red pentacle cells stand for dentritic cells and green pentacle ones for follicular dentritic cells. **(B)** At the T-B border, B cells receive signals from cognate CD4^+^ T cells to activate and proliferate. **(C)** Within the germinal centers (GCs), MOG-specific B cells recognize antigen and present it to Tfh cells. After receiving activation signal of MHC-peptide complex and ICOSL from B cells, Tfh cells secret large amount of IL-21 and express CD40L. IL-21 and CD40L bind to the relevant receptor and ligand on B cells, respectively. The ligation of CD40L and CD40 promote the stabilization of NF-κB-inducing kinase (NIK), and activate the downstream pathway, which lead the formation of P52 and initiate the expression of B lymphocyte-induced maturation protein-1 (Blimp-1). IL-21 receptor activates the JAK/STAT3 pathway and phosphorylated STAT3 (p-STAT3) promotes the expression of Blimp-1. Moreover, P-STAT3 could also upregulate the expression of NIK, probably through stimulating the *de novo* synthesis of NIK (red thin arrow). Thus, the signal provided by IL-21 and CD40L synergistically facilitates the differentiation of B cells and autoantibody production.

B cells need help from CD4^+^ T cells to undergo activation, proliferation, and differentiation. Outside GCs, activated CD4^+^ T cells, mainly Th1, Th2, or Th17 cells, stimulate naïve B cells to activate and proliferate through CD40L. Only in GCs, Tfh cells help B cells differentiate into plasma cells by expressing IL-21 and CD40L (Figure 7A). Although IL-21 and CD40L have been identified as the major help molecules for Tfh cells to induce B cell activation and differentiation (43), the intracellular mechanism responsible for this function is poorly understood. The present findings offer novel insight regarding the synergistic effect between IL-21 and CD40L in boosting antibody production. Such an effect may be mediated by potential crosstalk between the JAK/STAT and non-canonical NF-κB pathways. Surprisingly, IL-21 slightly enhanced the expression of NIK, a key kinase of the non-canonical NF-κB pathway. This upregulated function was blocked by the phosphorylation inhibitor of STAT3, consistent with the results reported by a previous study (44). A previous chip-sequencing analysis has identified candidate STAT3 binding sites in the promoter of the murine *NIK* locus (45). Thus, the research data above suggest that p-STAT3 might promote the synthesis of NIK protein. NIK is consistently ubiquitinated intracellularly by TRAF-cIAP E3 ubiquitin ligase complex, when cells are not exposed to inducers of the non-canonical NF-κB pathway (46). Activation of NIK is the rate-limiting step in the non-canonical NF-κB pathway, which is predominantly controlled by the stabilization and *de novo* synthesis of NIK protein. Results from the present work suggest that, when B cells are co-treated with IL-21 and CD40L, *de novo* synthesis of NIK is enhanced by p-STAT3. More importantly, CD40–CD40L interaction stabilizes NIK and activates the non-canonical NF-κB pathway (Figures 7B,C). Co-activation of the JAK/STAT and non-canonical NF-κB pathways can upregulate Blimp-1, a key transcription factor of plasma cells. Taken together, IL-21 and CD40L function in concert to stimulate the differentiation of B cells and antibody production. The biochemical events within the B cells during the differentiation phase remain ambiguous. Another study also showed the synergy of IL-21 and CD40L, which promoted the differentiation of human plasma cells (43). They found CD40L–CD40 ligation greatly enhanced the signaling of JAK/STAT3, and IL-21 and CD40L collaborated to promote the expression of Blimp-1 at chromatin level. However, in our study, we did not observe the facilitating effect of CD40 signal to JAK/STAT3. Our study revealed a novel mechanism of the synergy between IL-21 and CD40L, which preliminarily provide the differentiation signals to B cells, while BCR provide the activation signal. More importantly, the pathogenicity of anti-MOG_35–55_ antibodies was also preliminarily explored in our research, namely that complement C3b deposited in the sections of spinal cords where infiltrated-B cells aggregated and the density of MOG protein significantly decreased, suggesting autoantibodies secreted by infiltrated-B cells activate classical complement pathway and complement-dependent cytotoxicity leads to demyelination.

Several previous studies have revealed that MOG_35–55_ peptide-induced model is B cell independent, as EAE could be also induced in B cell-deficient mice using MOG_35–55_. This indicated that B cells were not necessary for induction of EAE. Our adoptive transfer experiments also confirmed this point, namely that adoptive transfer indeed did not advance the disease onset of EAE mice. Nevertheless, in our study, adoptive transfer could obviously delay the remission of EAE, which suggested that B and Tfh-like cells might contribute to the pathogenesis at late stages in this model. Amplification of Tfh and B cells may be an important cause of the continued deterioration of the disease, but these two types of cells might not be essential factors of the disease onset. We suspect that this may be caused by the following reasons. On the one hand, B and Tfh cells might enhance Th17 and Th1 response. A recent study has shown that Tfh cells promoted Th17-induced neuroinflammation, while Tfh cells alone could not effectively induce EAE (47). On the other hand, Tfh cells enhance the antibodies production by B cells, which may further increase the antibody-mediated cytotoxicity. Moreover, as Flach et al. illustrated, myelin-specific autoantibodies could concentrate myelin antigens in CNS-resident phagocytes, which consequently increase their capacity to present autoantigen (15).

In conclusion, though EAE is presently perceived to be dominated by Th1 and Th17 cells, the present results suggest that Tfh-like cells participate in the pathogenesis of EAE by facilitating the production of anti-MOG_35–55_ antibody and forming ELSs and GC-like structure in the CNS. More importantly, the level of circulating Tfh-like expression is correlated with disease activity in MS patients. These findings support that Tfh-like cells and its related functional molecules are potential biomarkers and therapeutic targets in MS. The definitive role contributed by Tfh cells in MS patients should be confirmed in future clinical studies.

## Ethics Statement

This study was carried out in accordance with the recommendations of “the Biomedical Research Guideline involving Human Participants, National Health and Family Planning Commission of China” with written informed consent from all subjects. All subjects gave written informed consent in accordance with the Declaration of Helsinki. The protocol was approved by the “Tangdu Hospital Ethical Review Board of Fourth Military Medical University.”

## Author Contributions

KY and HL designed the study. JG immunized the mice and performed the immunofluorescence staining. CZhao performed western blotting analysis and cell-coculture experiment. FW and CZhang performed the flow cytometry. LT and YS performed the CLISA. SY, DJ, and JW took care of the mice. DZ took care and followed up the patients with MS. ZL and HL diagnosed RR-MS patients. JG and CZhao drafted the manuscript and figures.

## Conflict of Interest Statement

The authors declare that the research was conducted in the absence of any commercial or financial relationships that could be construed as a potential conflict of interest.
