# Supplementary material for: T Follicular Helper-Like Cells Are Involved in the Pathogenesis of Experimental Autoimmune Encephalomyelitis
Source: Front Immunol. 2018 May 7;9:944. doi: 10.3389/fimmu.2018.00944 (PMC5949363; doi:10.3389/fimmu.2018.00944)
Supplement: Supplementary file 1 [file Data_Sheet_1.docx]

**T Follicular Helper-Like Cells Are Involved in the Pathogenesis of Experimental Autoimmune Encephalomyelitis**

*Jun Guo^1†^, Cong Zhao^1,2,3†^, Fang Wu^1,2,4†^, Liang Tao^2^, Chunmei Zhang^2^, Daidi Zhao^1^, Shuya Yang^2^, Dongbo Jiang^2^, Jing Wang^2^, Yuanjie Sun^2^, Zhuyi Li^1^, Hongzeng Li^1*^ and Kun Yang2^*‡^*

*^1^Department of Neurology, Tangdu Hospital, Fourth Military Medical University, Xi’an, China, ^2^Department of Immunology, Fourth Military Medical University, Xi’an, China, ^3^Department of Neurology, Air Force General Hospital PLA, Beijing, China, ^4^Department of Neurology, Xi’an Children’s Hospital, Xi’an, China*

**Supplementary Material**


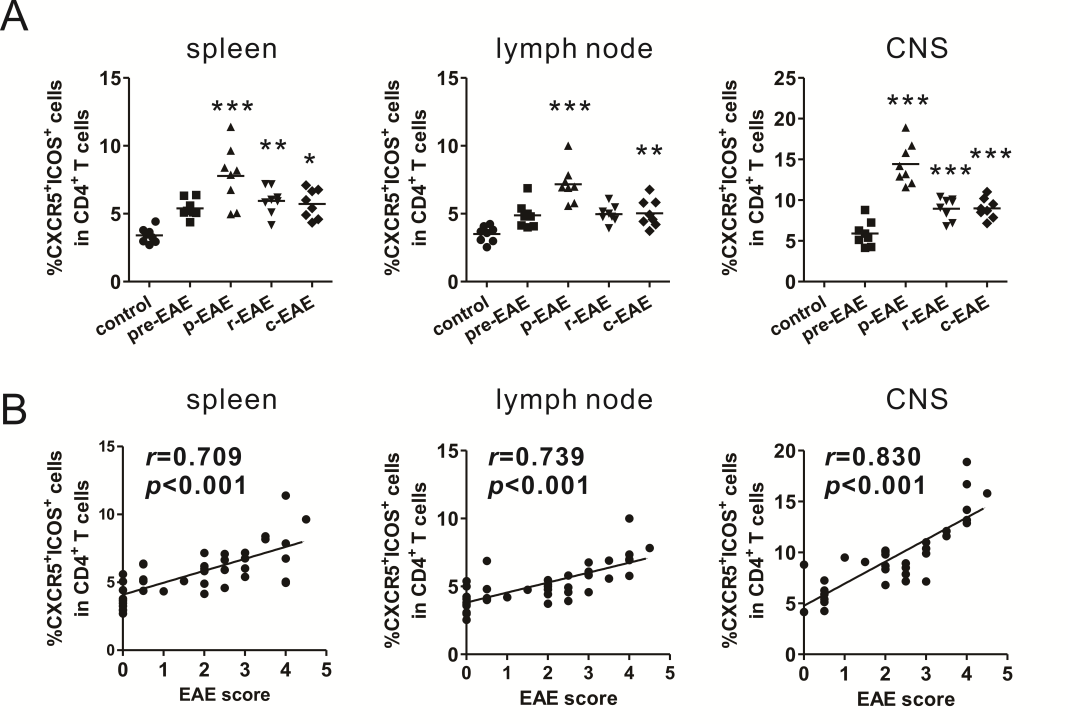


**Supplementary Figure 1** Kinetics of T follicular helper (Tfh)-like cell in the secondary lymphoid organs (SLOs) and the central nervous system (CNS) during the different phases of experimental autoimmune encephalomyelitis (EAE). **(A)** Comparisons of the frequency of CD4^+^CXCR5^+^ICOS^+^ Tfh-like cells in the spleens, draining lymph nodes and CNS at different phases of EAE mice and control mice. Each data point represents an individual subject and the horizontal lines represent the means (*n* = 8/time point). **P*<0.05, ***P*<0.01, ****P*<0.001 vs. control. **(B)** The correlation analysis between the frequency of CD4^+^CXCR5^+^ICOS^+^ Tfh-like cells and EAE score. Results are representative of three independent experiments.


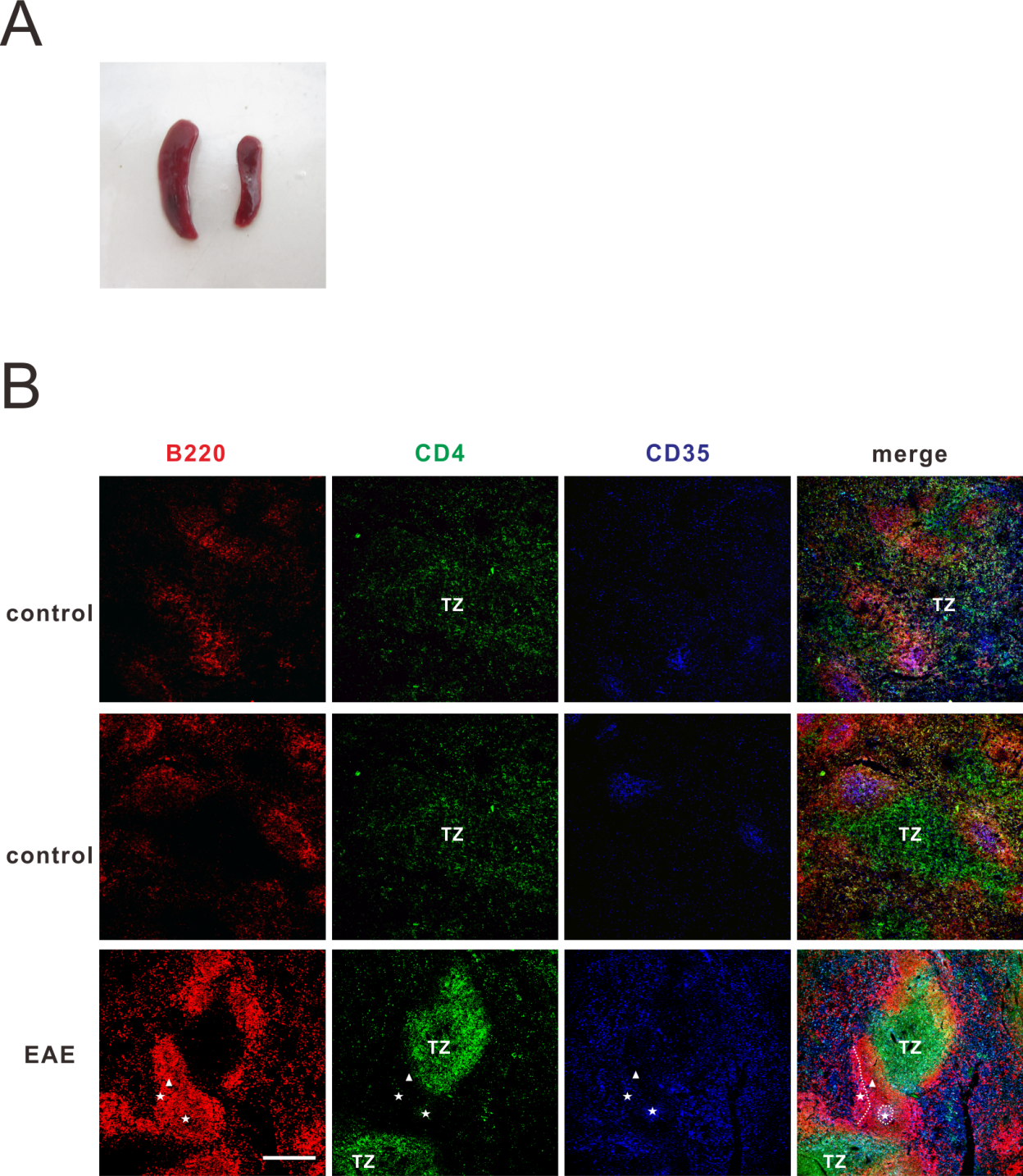


**Supplementary Figure 2** Comparisons of splenic specimens and the GC structures between the spleen of control and EAE mice. **(A)** The spleen of EAE mice at the peak phase (left) and the spleen of the control mice (right). **(B)** Immunofluorescence staining compared the GC structures between the spleen of control and EAE mice (TZ = T-cell zone, white triangle: dark zone, white pentacle: light zone, bar = 200μm). Sections are representative of three mice analyzed.


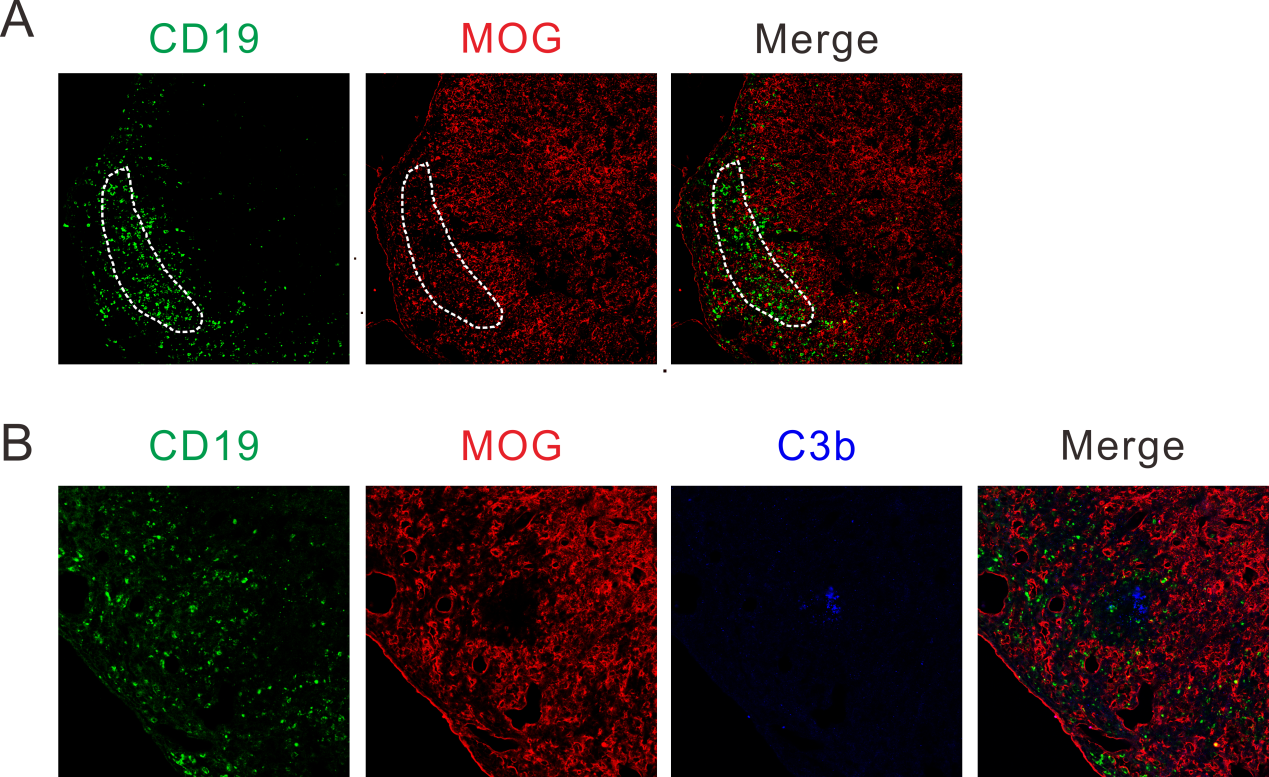


**Supplementary Figure 3** **(A)** Immunofluorescence staining showed that the density of MOG protein significantly reduced at the place where B cells aggregated. **(B)** Immunofluorescence staining showed that the density of MOG protein significantly reduced at the place where complement C3b deposited.


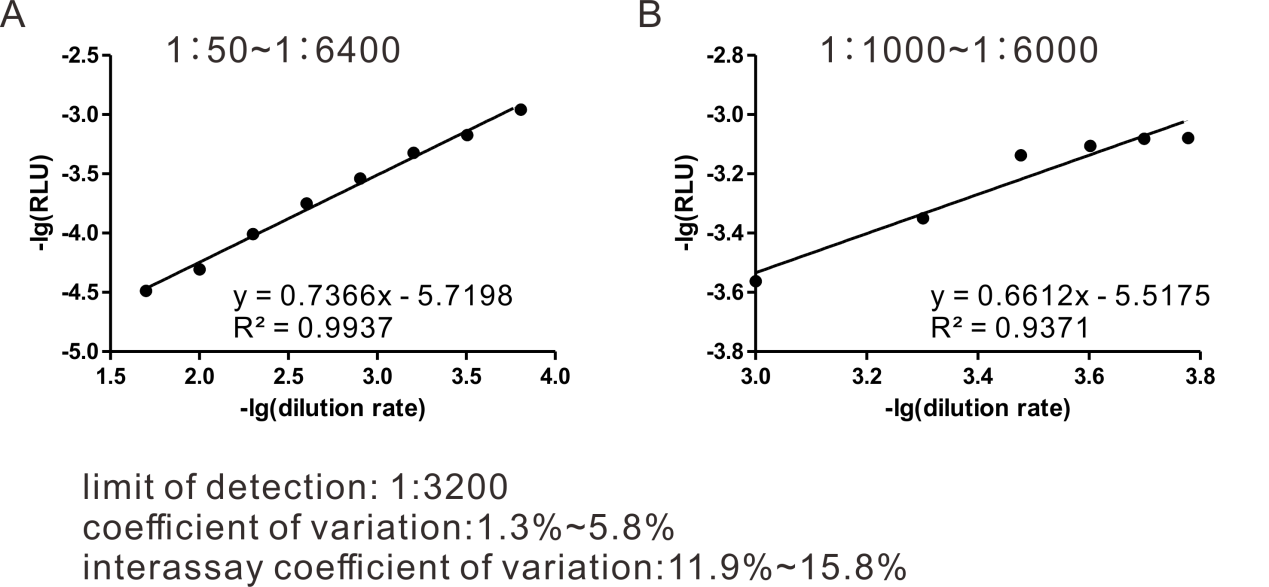


**Supplementary Figure 4** A novel and sensitive chemiluminescent enzyme-linked immunosorbent assay (CLISA) to detect anti-MOG_35-55_ antibodies established in this research. **(A)** The linear relation between –lg(dilution rate) and –lg(RLU). Dilution rates of the serum sample include 1/50, 1/100, 1/200, 1/400, 1/800, 1/1600, 1/3200, and 1/6400. **(B)** The linear relation between –lg(dilution rate) and –lg(RLU). Dilution rates of the serum sample are 1/1000, 1/2000, 1/3000, 1/4000, 1/5000, and 1/6000.


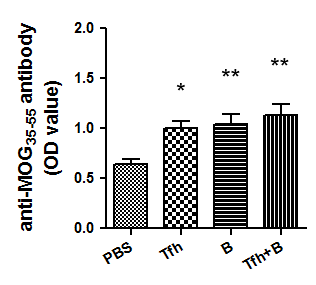


**Supplementary Figure 5** Detection of anti-MOG_35-55_ in recipient mice. All the sera were collected at 15 dpi from the tail vein. Sera were used for antibody detection after 1:2 dilution. All data were shown as mean ± SEM. * *P*<0.05, ** *P*<0.01 vs. PBS. Results are representative of three independent experiments.
